# Progress and application of adipose-derived stem cells in the treatment of diabetes and its complications

**DOI:** 10.1186/s13287-023-03620-0

**Published:** 2024-01-02

**Authors:** Dongxu Yan, Yujie Song, Bing Zhang, Guojie Cao, Haitao Zhou, Hong Li, Hao Sun, Meng Deng, Yufeng Qiu, Wei Yi, Yang Sun

**Affiliations:** 1 Department of General Medicine, Xijing Hospital, Fourth Military Medical University, 127# Changlexi Road, Xi’an, 710032 China; 2 Department of Cardiovascular Surgery, Xijing Hospital, Fourth Military Medical University, 127# Changlexi Road, Xi’an, 710032 China

**Keywords:** Adipose-derived stem cells, Cell therapy, Diabetes mellitus, Complications, Diabetic wounds, Retinopathy, Nephropathy

## Abstract

Diabetes mellitus (DM) is a serious chronic metabolic disease that can lead to many serious complications, such as cardiovascular disease, retinopathy, neuropathy, and kidney disease. Once diagnosed with diabetes, patients need to take oral hypoglycemic drugs or use insulin to control blood sugar and slow down the progression of the disease. This has a significant impact on the daily life of patients, requiring constant monitoring of the side effects of medication. It also imposes a heavy financial burden on individuals, their families, and even society as a whole. Adipose-derived stem cells (ADSCs) have recently become an emerging therapeutic modality for DM and its complications. ADSCs can improve insulin sensitivity and enhance insulin secretion through various pathways, thereby alleviating diabetes and its complications. Additionally, ADSCs can promote tissue regeneration, inhibit inflammatory reactions, and reduce tissue damage and cell apoptosis. The potential mechanisms of ADSC therapy for DM and its complications are numerous, and its extensive regenerative and differentiation ability, as well as its role in regulating the immune system and metabolic function, make it a powerful tool in the treatment of DM. Although this technology is still in the early stages, many studies have already proven its safety and effectiveness, providing new treatment options for patients with DM or its complications. Although based on current research, ADSCs have achieved some results in animal experiments and clinical trials for the treatment of DM, further clinical trials are still needed before they can be applied in a clinical setting.

## Background

Diabetes mellitus (DM) is an endocrine disease that is prevalent worldwide, and its incidence has been rapidly increasing in recent years. According to the World Health Organization, DM is expected to affect over 693 million people by 2045 [1]. DM is a metabolic disorder that is marked by either absolute or relative insulin deficiency or insulin resistance, resulting in a clinical syndrome that manifests as a range of symptoms [2]. In addition, DM is a systemic vascular disorder that occurs when metabolic irregularities in the body cause glucose buildup, leading to a range of diabetic vascular complications, including chronic microvascular disease and macrovascular atherosclerosis [3, 4]. The disease can affect multiple organs and systems in the body and it is a leading cause of premature death [5]. The occurrence of DM is caused by the combined effects of genetic and environmental factors [6, 7]. DM is a complex condition that can be broadly categorized into four types based on its etiology: type 1 diabetes mellitus (T1DM), type 2 diabetes mellitus (T2DM), gestational diabetes, and other types of diabetes [8]. Currently, the treatment of DM primarily relies on administration of exogenous insulin or oral intake of hypoglycemic drugs. However, these methods can only alleviate the symptoms of patients and cannot cure DM completely [9]. Therefore, once diagnosed with DM, patients need to continuously take medication or use insulin to control blood sugar, alleviate symptoms, and prevent disease progression [10]. This has a multifaceted impact on the daily life of patients, not only requiring them to constantly monitor the side effects and risks of hypoglycemic drugs but also inflicting a heavy economic burden to their families and society [11]. Therefore, exploring alternative treatments for DM and maintaining their therapeutic effects is necessary.

Various diseases can be treated with stem cells because of their ability to self-renew, differentiate into other cell types, and regulate immunity. There is an ongoing ethical debate surrounding the procurement and utilization of embryonic stem cells [12]. Compared with other stem cells, the adipose-derived stem cells (ADSCs) have wider and more convenient sources, such as adipose tissue in the abdomen, limbs, and face areas and obtaining ADSCs only causes minor damage [13, 14]. Animal models of DM have been treated with ADSCs, and some human clinical trials (phase I/II) have also utilized these cells [15], with a few even progressing to phase III trials [16]. This article focuses on the research progress of ADSCs in the treatment of DM and its complications and explores its underlying mechanisms of action.

### ADSCs

In recent years, there have been experiments using mesenchymal stem cells (MSCs) from different sources to treat diabetes. The early focus was on bone marrow-derived MSCs (BM-MSCs), which contain various types of stem cells including hematopoietic stem cells, mesenchymal stem cells, and endothelial progenitor cells. After the application of BM-MSCs, the patient’s insulin requirement decreased, insulin sensitivity increased, and β-cell function improved [17–19]. However, BM-MSCs are obtained invasively through the femur or iliac bone, which is not only painful but also yields a small quantity of cells and carries the risk of infection after extraction [20]. Umbilical cord-derived MSCs have a higher similarity to embryonic stem cells and possess greater differentiation potential compared to other common types of MSCs. After the infusion of Umbilical cord-derived MSCs, there was an increasing trend in the number of regulatory T (Treg) cells and a slight decrease in insulin requirements [21]. Jiang et al. [22] studied the use of placenta-derived MSCs in treating type 2 diabetes mellitus (T2DM), which resulted in a decrease of ≥ 50% in insulin requirements and improvement in kidney and heart function to some extent. However, MSCs derived from fetal appendages are obtained after birth, which poses potential risks of allogeneic stem cells and corresponding ethical issues [23]. Following BM-MSCs and fetal appendage-derived MSCs, ADSCs have become an alternative choice for clinical cell therapy due to their easy accessibility, abundant source, subcutaneous location, and longer culture time [24, 25]. Compared with other types of MSCs, such as BM-MSCs, ADSCs have similar proliferation and differentiation abilities and can be obtained with less pain [26, 27]. The cell surface molecules of ADSCs have been found to include differentiation clusters such as CD9, CD29, CD36, CD44, CD49d, CD49e, CD51, CD55, CD73, CD82, CD105, CD106, CD271, and von Willebrand factor (vWF), among which CD36 and CD49d are unique to ADSCs, whereas CD3, CD11a, CD11c, CD31, CD33, CD45, CD133, c-Kit, Lin, major histocompatibility complex II, and human leukocyte antigen (HLA)-DR surface proteins are lacking [28].

In some experiments, ADSCs showed genetic and epigenetic stability [29] and did not show significant immune response [30] and tumorigenicity [31]. Additionally, there have been no significant safety issues observed when ADSCs are transplanted into animal models [16, 29] or when used in human clinical trials [32]. According to existing research, ADSCs have not shown apparent safety issues and have a low potential for stimulating anti-HLA immune responses. There have been few reports of adverse reactions to ADSC-based treatments, but the long-term immunogenicity effects still need to be considered [33]. At the same time, in some studies, researchers have observed pulmonary embolism and infarction after injecting ADSCs into mice or patients [34, 35]. Other literatures have also reported an increase in levels of thrombin-antithrombin and D-dimer after intravenous infusion of allogeneic ADSCs, both of which are markers of coagulation activation [36].

Several experiments have explored the functions of ADSCs in different cell lineages [37, 38]. The advantages of ADSCs in cell replacement therapy and cell repair functions have been validated using animal models [39]. Some studies have demonstrated that direct intravenous or in situ injection of ADSCs restores the vitality of transplanted cells, which then differentiate and integrate ADSC functions in the body [40]. Transplanted ADSCs secrete several repair molecules such as neurotrophic factors [41], chemokines [42], immune regulatory factors [43], and inflammation regulatory factors [44]. To date, the therapeutic ability of ADSCs for diseases has been verified in several clinical trials [16, 38, 45–47]. These findings provide evidence of the safety of ADSCs and their potential in regenerative medicine, suggesting that ADSCs hold promise for use in human clinical trials.

## Application of ADSCs in type 1 diabetes mellitus

In T1DM, due to autoimmune reactions, T helper 1 (Th1) cells attack pancreatic β cells, leading to the loss of insulin-producing cells (IPCs) [48]. The presence of macrophages, dendritic cells, natural killer cells, and lymphocytes also accelerates the progression of T1DM [49]. CD4^+^ T cells and inflammatory factors, including interferon-gamma (IFN-γ), interleukin-2 (IL-2), and tumor necrosis factor-alpha (TNF-α), play important roles in the process of β cell damage [50]. As pancreatic β cells are the sole producers of insulin in the body, their death leads to a complete absence of insulin secretion, ultimately resulting in the development of diabetes [51].

Long-term complications, such as vascular degeneration, renal failure, and blindness, cannot be prevented with the current interventions. The methods that have been applied in clinical practice to replace β cells mainly include whole pancreas and islet cell transplantation [52]. However, there are still many obstacles to the development of these methods, such as a lack of suitable islet donors, the need for lifelong immunosuppressive therapy after transplantation, and the exhaustion of transplanted organs and cells in diabetic patients, all of which restrict the development of this technology [53]. In recent years, ADSCs have gradually gained the attention of researchers owing to their ability to self-renew, differentiate into other cell lineages, and regulate the immune system. It is hoped that the characteristics of ADSCs can be utilized to achieve the goal of curing T1DM.

## Animal models and human clinical trials

In the application of ADSCs to animal models, undifferentiated ADSCs or differentiated IPCs from ADSCs can be transplanted via intravenous, intraperitoneal, or renal capsule injection [44, 50, 54–61], as shown in Table 1. According to research, the mortality rate of mice within 24 h is close to 85% when a large number of ADSCs are administered through the tail vein. Reducing the injection quantity can avoid similar occurrences [35]. Some studies use intraperitoneal or renal capsule injection to attempt to avoid this problem [50, 55, 57, 58]. Currently, the use of ADSCs for treating T1DM patients is still in the preliminary research stage. There are not many studies related to this direction, and there are differences in administration routes. Currently, the administration routes that have been used include differentiating ADSCs into IPCs and then injecting them into the portal vein, thymus, or subcutaneous tissue [62]. Alternatively, ADSCs can be induced to become insulin-secreting ADSCs and co-transplanted with unfractionated cultured bone marrow cells into the portal vein of diabetic patients [15, 63]. It is administered through portal vein infusion because it allows the cells to stay in the liver microcirculation, and the liver, being a tolerant organ, would not reject cell engraftment. However, there is currently no consensus on the specific administration method that can achieve better therapeutic effects. In addition, regarding the number of transplanted ADSCs, there are also variations in current studies. Some studies used a level of 10^6^ cells, while others used a level of 10^8^ cells, as shown in Table 2 [15, 62, 63]. However, the specific number of cells required to achieve therapeutic effects while minimizing the potential risks of ADSCs still needs further investigation.

**Table 1:** Animal experiments of application of ADSCs in T1DM

Model	Sources	Type of cells	Admin /dosage (number of cells)/interval	Detections	Improvements
STZ-induced rats [54]	Human ADSCs	N.M	1. Intravenous 2. 2 × 10^6^ udADSCs 3. Once	1. Glucose↓ 2. Insulin↑	1. ADSCs improved impaired glucose tolerance, pancreatic morphology, and cell number in non-obese mice induced by STZ 2. Islet survival rate improved after co-culture with human ADSCs 3. Human ADSCs secreted sufficient VEGF and TIMP-1 4. ADSCs reduced cell death through combined local paracrine secretion
STZ-induced rats [55]	Rat ADSCs	CD54^+^, CD90^+^, MHC^+^, CD45^−^, MHC class II-	1. Left renal capsule 2. Islet (100 mg/kg) 3. Once	1. Glucose↓ 2. Insulin↑ 3. C-peptide↑	1. Co-culture of pancreatic islet cells and mesenchymal stem cells resulted in higher differentiation efficiency 2. Co-transplantation of pancreatic islet cells and mesenchymal stem cells led to better recovery compared to simple pancreatic islet transplantation 3. Co-transplantation of pancreatic islet cells and mesenchymal stem cells increased the number of insulin-producing cells
STZ-induced Sprague–Dawley rats [44]	Rat ADSCs	CD29^+^, CD90^+^, CD34^−^, CD31^−^, CD45^−^, CD13^−^	1. Intravenous 2. 1 × 10^7^ udADSCs 3. Consecutive days	1. Glucose↓ 2. Insulin↑ 3. Cholesterol↓ 4. Triglycerides↓ 5. Urea nitrogen↓ 6. Creatinine↓	1. Autologous ADSCs alleviated kidney damage induced by STZ 2. ADSC transplantation reduced oxidative stress in the T1DM model animal induced by STZ 3. ADSC transplantation reduced the levels of TNF-α, IL-1, and IL-6 in diabetic kidneys 4. ADSC transplantation inhibited the activation of the MAPK signaling pathway induced by STZ
STZ-induced Balb/c mice [64]	Rat ADSCs from 4 to 6 weeks of Sprague–Dawley rats	CD29^+^, CD90^+^, CD34-, CD31-	1. Intravenous 2. 1 × 10^6^ udADSCs 3. Once	1. Glucose↓ 2. Insulin↑	1. ADSCs reduced fasting blood glucose levels induced by STZ 2. ADSCs alleviated pancreatic damage caused by STZ 3. ADSCs increased insulin secretion from damaged islets induced by STZ
NOD (H2-A^g7^mice) [50]	Mice epididymal fat tissue from 8 weeks Balb/c mice	CD105^+^, CD90^+^, CD44^+^, CD73^+^, CD45^−^, CD11c^−^, CD11b^−^, CD34^−^	1. Intraperitoneal injection 2. 1 × 10^6^ udADSCs 3. Days 0, 7, and 14	1. Insulin↑ 2. Amylin↑ 3. Glucagon-like peptide1↑ 4. Interferon-γ↓ 5. CD4^+^↓ 6. Transforming growth factor-β1↑	1. By attenuating the Th1 immune response, the pathogenesis of autoimmune diabetes in NOD mice was effectively improved 2. Accompanied by the expansion/proliferation of Tregs 3. Helped maintain the function of β cells
NOD mice [57]	Epididymal and subcutaneous fat of anterior abdominal wall of mice	CD29^+^, CD44^+^, CD90^+^, CD105^+^, Sca-1^+^, CD80-, MHC class II-, CD45-	1. Under the kidney capsule 2. 400 donor islets or 800 IPCCs 3. Once	N.M	1. Blocked the death pathway and co-stimulation of CD28/CD40 2. It is necessary for the long-term survival of IPCs differentiated from ADSCs
STZ-induced mice [58]	Human ADSCs	CD90^+^, CD44^+^, CD29^+^, CD13^+^, CD59^+^, CD105^+^	1. Peritoneal cavity 2. 1,000–1,200 day 10 ICAs 3. Once	1. Glucose↓ 2. C-peptide↑	1. The mice maintained lower blood sugar levels over the following 8 weeks compared to mice treated with undifferentiated human ADSCs
STZ-induced mice [59]	Human ADSCs	CD29^+^, CD44^+^, CD71^+^, CD90^+^, CD105/SH2^+^, CD73/SH3^+^,	1. Tail vein injection 2. 5 × 10^5^ Pdx1-transduced ADSC 3. Once	1. Insulin↑ 2. C-peptide↑	1. ADSCs transduced with Pdx1 differentiated into IPCs in vivo 2. Pdx1-transduced ADSCs were stably transplanted into the pancreas, acquired a functional β-cell phenotype, and partially restored pancreatic function in vivo
STZ-induced rats [60]	Human and rat ADSCs	Stor-1^+^, CD34-	1. Under renal capsule 2. 2 × 10^6^ Insulin-producing ADSCs 3. Once	1. Glucose↓ 2. C-peptide↑	1. Handling group mice resulted in lower blood glucose levels and higher glucose tolerance 2. Histological examination revealed that transplanted cells formed tissue-like structures and expressed insulin
STZ-induced mice [61]	Human eyelid ADSCs	Oct-4, Rex1, SCF	1. Under the left renal capsule 2. 1.5 × 10^6^ Insulin-producing ADSCs 3. Once	1. Glucose↓ 2. Insulin↑ 3. C-peptide↑	1. Out of 20 receptor mice, 10 were normalized from hyperglycemia 2. Only human insulin and C-peptide were detected in the blood of receptor mice
STZ-induced Balb/c nude mice [65]	Human ADSCs	N.M	1. Under renal capsule 2. 2 × 10^6^ Pdx1-transduced ADSCs 3. Once	1. Glucose↓ 2. Insulin↑	1. The body secreted insulin according to the blood glucose level, reducing the blood glucose level
STZ-induced mice [66]	Human ADSCs	CD73^+^, CD90^+^, CD105^+^, CD14^−^, CD45^−^	1. Tail vein injection 2. 1 × 10^6^ udADSCs 3. Once	1. Glucose↓ 2. Insulin↑	1. Transplanted ADSCs promoted proliferation and insulin release of co-cultured pancreatic islets 2. The treatment group showed a significant reduction in blood glucose levels compared to the control group
STZ-induced rats [67]	Rat ADSCs	CD90^+^, CD73^+^, CD105^+^, CD44^+^, CD 45^+^, CD14	1. Splenic intravenous injection 2. 1.5 × 10^6^ IPCs 3. Once	1. Glucose↓ 2. Insulin↑	1. IPCs expressed β-cell markers and secreted insulin 2. Residual islets underwent diffuse proliferation leading to increased serum insulin levels 3. The blood glucose levels of the treatment group returned to normal

*ADSC* adipose-derived stem cells; *CD* cluster of differentiation; *ICAs* islet-like cell aggregates; *IL* interleukin; *IPCs* insulin-producing cells; *MHC* major histocompatibility complex; *N.M.* not mentioned; *NOD* non-obese diabetic; *Oct4* octamer-binding transcription factor 4; *Pdx1* pancreatic and duodenal homeobox 1; *Rex1* reduced expression protein 1; *SCF* stem cell factor; *STZ* streptozotocin; *T1DM* type 1 diabetes mellitus; *TNF-α* tumor necrosis factor α; *TIMP1* tissue inhibitor of metalloproteinase 1; *Th1* type 1 T helper; *Tregs* regulatory T cells; *udADSCs* undifferentiated ADSCs; *VEGF* vascular endothelial growth factor

**Table 2:** Clinical trials using ADSCs in T1DM

Model	Sources	Type of cells	Admin/dosage (number of cells)/interval	Detections	Improvements	Observed complications/adverse effects
Patients with T1DM [62]	Human ADSCs and hematopoietic cells	CD90^+^, CD73^+^, CD45-	1. Intraportal injection 2. 1.75 × 10^8^ IPCs 3. Once	1. Glucose↓ 2. C-peptide↑ 3. Glycosylated hemoglobin↓ 4. Glutamic acid decarboxylase↓	1. The average glycated hemoglobin level of the treatment group patients decreased from 10.99% to 6.72% 2. The average level of glutamic acid decarboxylase in the treatment group patients decreased from 331.10 IU/ml to 123 IU/ml	No untoward effects were observed
Patients with T1DM [15]	Human ADSCs	CD90^+^, CD73^+^, CD45^−^	1. Intraportal injection 2. 2.025 × 10^6^ insulin-secreting ADSCs 3. Once, days 0	1. Glucose↓ 2. C-peptide↑ 3. Glycosylated hemoglobin↓ 4.Exogenous insulin requirement↓	The exogenous insulin demand for patients in the treatment group decreased to 0.63 U/kg/day, and the average glycated hemoglobin level decreased to 7.39%. The C-peptide level in the serum increased to 0.37 ng/ml	There was no adverse/untoward side effect related to stem cell infusion or administration of induction therapy
Patients with T1DM [63]	Human ADSCs	CD90^+^, CD73^+^, CD45-	1. Intraportal injection 2. 1.41 × 10^6^ insulin-secreting ADSCs 3. Once, days 0	1. Glucose↓ 2. C-peptide↑ 3. Glycosylated hemoglobin↓	During a 2.9-month follow-up, patients in the treatment group had a 30%–50% reduction in insulin requirements and a 4–26-fold increase in serum C-peptide levels	There were no adverse side effects related to the stem cell infusion or the administration of induction therapy

*ADSC* adipose-derived stem cells; *CD* cluster of differentiation; *IPCs* insulin-producing cells; *T1DM* type 1 diabetes mellitus

## Mechanism of ADSCs in T1DM

### ADSCs differentiation to IPCs

According to current research, one of the mechanisms for using ADSCs to treat T1DM is to transplant differentiated IPCs or ADSCs into animals or humans and utilize their ability to secrete insulin. Timper et al. [68] conducted the first experiment to differentiate human ADSCs into IPCs. The glucagon-like peptide-1 (GLP-1) was used in another study to induce differentiation of human ADSCs into IPCs. Insulin and C-peptide were released by IPCs in a glucose concentration-dependent manner [61]. Additionally, the injection of differentiated IPCs or undifferentiated ADSCs into diabetic animals resulted in a rise in insulin levels in diabetic animal serum and a return to normal blood glucose levels, as shown in Table 1.

The differentiation of ADSCs into IPCs is influenced by many factors, including the Wnt signaling pathway. Pancreatic development, islet function, and insulin production and secretion depend on this pathway [69]. Some studies have found that activating the Wnt signal can induce ADSCs of rats to differentiate into IPCs, which can be identified by detecting the expression levels of genes such as *INS* (insulin), pancreatic and duodenal homeobox 1 (*PDX1*), and *GLP-1*, as well as the protein expression levels of PDX1, cytokeratin 19, nestin, insulin, and C-peptide [70]. Additionally, the phosphatidylinositol 3-kinase (PI3K)/Akt signaling pathway is also crucial in the differentiation of IPCs. The PI3K/Akt signaling pathway is significantly activated during the differentiation process of ADSCs into IPCs, mediated by the stromal cell-derived factor 1α and basic fibroblast growth factor [71]. According to a recent study, the upregulation of miR-375 is a crucial aspect of ADSC differentiation into IPCs [72]. A further aspect of its expression is that it is related to the secretion of insulin as well as cell proliferation [73]. Finally, IPCs are also developed through the Sonic hedgehog signaling pathway. To promote the development of IPCs, it is necessary to remove the inhibition of the Sonic hedgehog signaling pathway on them [74].

### Restore the function of residual pancreatic islets in the body

ADSCs can not only serve as a source of IPCs, but also support the function of residual pancreatic islets in patients with diabetes [75]. In these experiments, it was observed that the function of residual pancreatic was restored after injection of ADSCs or IPCs differentiated from ADSCs [50, 54, 59, 64, 66, 67, 76]. These transplanted ADSCs release a variety of cytokines, including interferon-induced protein 10, eosinophil chemotactic factor (eotaxin), vascular endothelial growth factor (VEGF), and tissue inhibitor of metalloproteinase 1 (TIMP-1), all of which can prevent apoptosis of β cells and promote β cell proliferation [54]. In addition, transplantation of ADSCs effectively improved the autoimmune mechanism of diabetes in non-obese diabetic mice by reducing the Th1 immune response and inducing the proliferation of Tregs to improve the high blood glucose levels of early-onset autoimmune diabetes [50]. Compared to the untreated diabetes group, the IPCs transplantation group showed increased pancreatic regeneration, as well as a significant increase in the number of islet cells, islet area and density, and C-peptide immunoreactive area. The percentage of collagen fiber area in the islets of the IPCs transplantation group also decreased [67].

### Maintain the function of pancreatic islet grafts in vivo or in vitro

ADSCs can also be used for preconditioning of pancreatic islet grafts in vitro to enhance the viability of the transplanted islets. Existing studies have shown that co-culturing ADSCs with syngeneic islets in vitro can significantly increase the level of insulin release compared to islets cultured alone. These pre-cultured pancreatic islet transplants have a higher success rate during transplantation and significantly improve the hyperglycemic condition in diabetic mice [54, 55, 76–79]. Eotaxin [54], VEGF [54, 77], TIPP-1 [54], extracellular matrix (ECM) components, annexinA1 [78] and fibroblast growth factor 2 expression [77] in ADSCs seem to be upregulated as a result of paracrine communication between pancreatic islets and ADSCs.

In addition to pre-treating pancreatic islet grafts, studies have attempted co-transplantation of ADSCs and islets to explore the effectiveness of this approach [55, 76, 80–82]. Adipose-derived stem cells (ADSCs) can promote the generation of a new vascular network within the co-transplanted islets by secreting various angiogenic factors, including VEGF [77, 83], hepatocyte growth factor [83], kinase insert domain receptor [83], transforming growth factor-beta (TGF-β) [78], and IL-8 [84]. ADSCs can also significantly inhibit the production of pro-inflammatory cytokines such as IFN-γ [78], TNF-α [76, 85], IL-6β [76], and IL-17 [78]. ADSCs can suppress the infiltration of CD4^+^ and CD8^+^ T cells [80] and macrophages [85], thereby reducing the inflammatory response within the co-transplanted islets. Co-transplantation of ADSCs and islets into STZ-induced diabetic mice significantly increased vascularization of the transplanted islets and significantly suppressed infiltration of inflammatory cells, resulting in increased survival time of the co-transplanted islets [80].

Successfully differentiating ADSCs into IPCs requires a specific combination of culture media, including insulin, transferrin supplement, and nicotinamide. This means that in clinical applications, a feasible method needs to be found to provide these media components. Although ADSCs can differentiate into IPCs, their insulin secretion capacity is still relatively low. Therefore, further optimization of the differentiation process is needed to improve the efficiency and functionality of ADSCs differentiating into IPCs. Further research is also needed to determine the transplantation method and the number of transplanted cells, which will contribute to the translation of ADSCs into a clinical therapeutic approach.

## Application of ADSCs in type 2 diabetes mellitus

T2DM is distinguished by insulin resistance in insulin-responsive tissues and impaired insulin secretion by pancreatic β cells. This type of diabetes accounts for 85–95% of all DM cases [86]. Additionally, excessive nutrition can lead to inflammation in adipose tissue, affecting multiple tissues and worsening insulin sensitivity and β cell function [86, 87].

Currently, there are few clinical trials on the treatment of T2DM with ADSCs. Most applications are still being tested in animal experiments, which indicate that injecting ADSCs through the tail vein, peritoneum, and renal capsule of mouse can improve hyperglycemia by restoring pancreatic β cells, reducing inflammation, and increasing insulin sensitivity [88, 89]. The exploration of some of these mechanisms has provided new directions for the clinical application of ADSCs [90–93]. See Table 3 for details.

**Table 3:** Animal experiments of application of ADSCs in T2DM

Model	Sources	Type of cells	Admin/dosage (number of cells)/interval	Detections	Improvements
STZ-induced mice [88]	Mice epididymal ADSCs	CD105^+^, CD29^+^, CD45^−^, CD34^−^	1. Tail vein injection 2. 5 × 10^5^ udADSCs 3. Once	1. Insulin↑ 2. C-peptide↑	1. Two weeks after the infusion of cells, T2DM mice receiving ADSCs cleared blood glucose more quickly during IPGTT compared to the T2DM group 2. Insulin sensitivity increased 3. Infusion of ADSCs improved the damaged islets and restored β cells in T2DM mice 4. Infusion of ADSCs reduced the size of adipocytes caused by HFD feeding
STZ-induced Sprague–Dawley rats [89]	Rats groin ADSCs	CD44^+^, CD105^+^, CD90^+^, CD73^+^, CD34^−^ , CD45^−^	1. Tail vein injection 2. 2 × 10^6^ udADSCs 3. Once	1. Glucose↓ 2. C-peptide↑ 3. Glycosylated hemoglobin↓ 4. Weight↓ 5. Insulin↑	1. The caspase-3 activity of the ADSC infusion group was significantly lower than that of the diabetes control group, but still higher than that of the normal control group 2. The vWF level of the ADSC infusion group was higher than that of the diabetes control and normal control groups, indicating that ADSCs can promote vascular reconstruction 3. ADSC infusion improved insulin sensitivity in diabetic rats
STZ-induced mice [91]	Mice ADSCs	CD90^+^, CD73^+^, CD105^+^, CD71^+^	1. Peritoneal cavity injection 2. 1000–1200 day 10 ICAs 3. Once	1. Glucose↓ 2. C-peptide↑ 3. Insulin↑	Four weeks after transplantation, ICAs taken from mice were still able to secrete C-peptide and insulin and reduce blood glucose levels in mice
STZ-induced Sprague–Dawley rats [93]	Rats ADSCs	N.M	1. Tail vein injection 2. 3 × 10^6^ udADSCs 3. Once	1. Glucose↓	1. Two days after injection of ADSCs, there was a significant decrease in blood glucose levels compared to the control group 2. From 3–24 h after injection of ADSCs, there was a slow decrease in blood glucose levels compared to the control group, and the expression of PI3K/p-AKT showed a decrease 3. Injection of ADSCs regulated the expression of liver glucose metabolism-related enzymes, possibly through increased AMPK phosphorylation
STZ-induced mice [94]	Human eyelid ADSCs	N.M	1. Under renal capsule 2. 1.5 × 10^6^ udADSCs 3. Once	1. C-peptide↑ 2. Insulin↑ 3. Glucose↓	1. The insulin and C-peptide levels in the ADSCs-treated group of mice were significantly higher compared to the untreated group 2. The triglyceride and IL-6 levels in the ADSC-treated group of mice were significantly lower compared to the untreated group 3. Human gene expression was observed in the kidney tissue of mice treated with ADSCs at both 60 and 210 days after treatment, while normal mouse kidney tissue showed no human gene expression
STZ-induced SD rat[95]	Rats ADSCs	N.M	1. Tail vein injection 2. 3 × 10^6^ udADSCs 3. Once a week for 24weeks	1. Glucose↓ 2. Serum lipid levels↓ 3. Insulin↑	1. Multiple ADSC infusions improved glucose homeostasis by improving insulin sensitivity and promoting pancreatic islet recovery in long-term T2DM complication rats 2. Multiple ADSC infusions attenuated T2DM-induced kidney damage, liver disease, lung disease and cardiac changes in long-term T2DM complication rats 3. Multiple ADSC infusions attenuated inflammation and changed the phenotypes of macrophages in the target organs of diabetic complications in long-term T2DM complication rats
STZ and immune-deficient mice [94]	Human eyelid ADSCs	HLA-DR-, HLA-DM-, CD80-, CD86-	1. Under renal capsule 2. 1.5 × 10^6^ IPCs 3. Once	1. Glucose↓ 2. C-peptide↑ 3. Insulin↑	1. Transplanted IPCs secreted insulin and C-peptide according to blood glucose levels, reducing blood glucose levels 2. Compared to the blank group, the transplantation of IPCs reduced IL-6, triglycerides, and free fatty acids in the body

*ADSC* adipose-derived stem cells; *AMPK* AMP-activated protein kinase; *CD* cluster of differentiation; *HFD* high-fat diet; *ICAs* islet-like cell aggregates; *IL* interleukin; *IPGTT* intraperitoneal glucose tolerance test; *N.M.* not mentioned; *PI3K* phosphatidylinositol 3-kinase; *STZ* streptozotocin; *T2DM* type 2 diabetes mellitus; *udADSCs* undifferentiated ADSCs; *vWF* von Willebrand factor; *N.M.* not mentioned

## Mechanism of action of ADSCs in T2DM

### Improvement of insulin resistance

Transplantation of ADSCs in vivo was discovered to restore the count of glucose transporter 4 and insulin receptors on the cell membranes of skeletal muscle, liver, and adipose tissue, and increase the phosphorylation of insulin receptor substrate 1 in a high-fat diet/STZ-induced T2DM rat model. Consequently, this alleviated the state of hyperglycemia and insulin resistance [89]. Insulin resistance may be linked to systemic chronic inflammation related to obesity, where inflammatory factors can impede the phosphorylation of the insulin receptor substrate and PI3K in the insulin signaling pathway, resulting in obstructed signal transduction and insulin resistance [96]. Studies have shown that after ADSCs are injected, TNF-α, IL-6, and IL-1β in T2DM rats are significantly reduced [89]. Injection of ADSCs reduces liver weight and fat degeneration by inhibiting the expression of pro-inflammatory genes and reduces the level of insulin resistance by increasing the expression of insulin receptor substrate [88], indicating that injection of ADSCs has a favorable effect on liver fat degeneration.

### Promotion of insulin production

In addition to insulin resistance, the dysfunction of pancreatic β cell also plays a crucial role in the development of T2DM. Animal experiments have shown that after different sources of ADSCs are transformed into IPCs in vitro and transplanted into mice, they can effectively reduce the blood glucose levels compared with injecting undifferentiated ADSCs and blank groups [91, 94]. ADSCs can facilitate the restoration of remaining pancreatic islet function and enhance the quantity of pancreatic islet β cells. They repair pancreatic islet cells by decreasing caspase-3 activity and promote pancreatic islet vascularization by secreting angiogenic factors such as VEGF, insulin-like growth factor 1, hepatocyte growth factor (HGF), and vWF, thus contributing to the regeneration of pancreatic islet β cells [89].

### Regulating liver glucose metabolism

Within 24 h after infusion of ADSCs, the hyperglycemic state of a T2DM rat model can be quickly relieved. This rapid action cannot be fully explained by improving β cell function and insulin resistance. The liver maintains normal blood glucose levels by regulating glycogen metabolism and gluconeogenesis. A 24-h period after ADSC infusion, T2DM rat models’ liver enzyme levels increased related to glucose metabolism, suggesting that ADSCs have a rapid effect on glucose homeostasis [93]. However, only this one research has reported similar phenomena, and the specific mechanism has not been clearly expounded.

However, it is worth noting that the clinical application of ADSCs in T2DM treatment is still in its early stages and requires further research to fully understand their safety and efficacy. Many technical and regulatory issues still need to be addressed. Despite these challenges, the potential benefits of ADSCs in T2DM treatment are significant, and their use represents a promising direction for future research and development in the field of regenerative medicine.

**Fig. 1:**
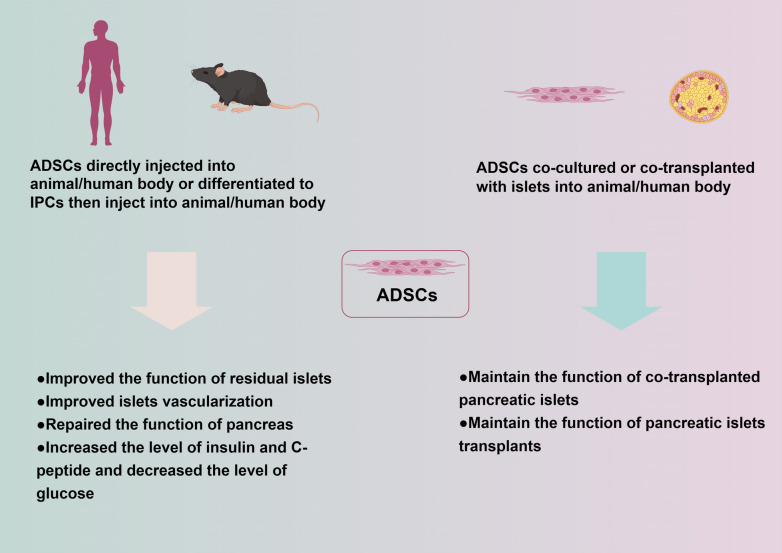
The application and mechanism of ADSCs in Diabetes Mellitus. *ADSCs* adipose-derived stem cells, *IPCs* insulin-producing cells. All of the elements in the diagram were provided by Figdraw (http://www.fgdraw.com)

The potential mechanisms of ADSCs application in DM are summarized in Fig. 1.

## Application of ADSCs in DM complications

### Diabetic wounds

Diabetic wounds are a chronic complication of DM, which severely affect the quality of life of patients with diabetes. Diabetic foot ulcer is the most serious form of diabetic wounds [97], which is clinically manifested as peripheral neuropathy and lower limb ischemia, leading to sensory disorders, muscle atrophy, rest pain, and necrosis [98]. Moreover, if diabetic wounds are not treated properly, they may lead to amputation or even death [99]. Peripheral neuropathy can decrease the skin elasticity and secretion function of diabetic wounds, making patients with diabetes more prone to form wounds on the skin [100]. At the cellular level, DM damages macrophage function and prevents keratinocytes and fibroblasts from playing their roles in epithelial healing [101]. In addition, hyperglycemia causes endothelial damage in peripheral blood vessels, which further reduces skin perfusion and promotes the formation of skin ulcers [101].

Currently, numerous animal and clinical human experiments have been carried out on the application of ADSCs in the treatment of diabetic wounds, as shown in Table 4 and Table 5.

**Table 4:** Animal experiments of application of ADSCs in diabetic wounds

Models	Sources	Admin/dosage (number of cells)/interval	Detections	Improvements
STZ-induced rats [102]	Human ADSCs	1. Peri-wound Injection 2. 3 × 10^6^ADSCs 3. 24 h after surgery	1. Gross morphology 2. Histology 3. Tissue VEGF	1. After 25 days, the recovery rate of rats treated with ADSCs was higher than that of the non-diabetic group (the non-treatment group of diabetic rats did not all survive until the end of observation) 2. 25 days after ADSC transplantation, the stem cells were still recognizable and dispersed in the muscle tissue, without structural differentiation 3. Three days after ADSC transplantation, the serum VEGF level was significantly higher than that of the non-diabetic and diabetic non-treatment groups
Ketamine- and xylazine-induced rats [103]	Human ADSCs	1. Collagen gel directly covering the wound 2. N.M 3. Immediately after surgery	1. Wound size 2. Histology of wounds	1. After 10 days of treatment, the wound size of the ADSC group was significantly smaller than that of the untreated group 2. Histological morphology of the wound showed that the scar dermis of the ADSC-treated group was thicker than that of the untreated group
STZ-induced rats [104]	Rat ADSCs	1. Peri-wound Injection 2. 1 × 10^6^ Rat ADSCs 3. Immediately after surgery	1. Wound closure area 2. Histology of wounds 3. Vessel density 4. Immunofluorescent analysis	1. The time for complete wound closure was significantly shortened in the ADSC treatment group 2. Histological observations showed that the tissue regeneration ability of the ADSC group was significantly higher than that of the control group 3. Immunofluorescence showed that ADSCs were incorporated into the regenerating epidermal structure and enhanced wound epithelialization 4. ADSC treatment significantly increased the formation of new blood vessels in the wound, and the vascular density was significantly increased compared to the control group
STZ-induced rats [105]	Rat SVF cells	1. Direct application of the reconstituted medium onto the wound 2. 5 × 10^5^ Rat SVF cells 3. Immediately after surgery	1. Cytokine levels 2. Cell amounts 3. Granulation tissue area 3. Vessel density	1. The granulation tissue of rats treated with SVF showed a relative increase in extension compared to untreated granulation tissue 2. The vascular density of rats treated with SVF showed a significant increase compared to untreated vascular density
Zucker diabetic obese rats [106]	Lewis rat and EGFP rat ADSCs	1. Cell sheet cover on the wound 2. N.M 3. N.M	1. Wound closure time 2. Vessel densities	1. The wound area and healing time of the transplantation group were superior to those of the non-transplantation group 2. The thickness of the connective tissue in the wounds of the transplantation group was significantly greater than that of the non-transplantation group 3. On experimental day 14, the vascular density of the transplantation group was approximately 2.5 times that of the non-transplantation group
STZ-induced rats [107]	Rat ADSCs	1. Peri-wound injection 2. 1 × 10^7^ Rat ADSCs 3. N.M	1. Wound size 2. Peri-wound inflammatory responses	1. The wound size in the ADSC transplantation group was significantly smaller than that in the non-transplantation group 2. Compared to the control group, the ADSC transplantation group inhibited the inflammatory response around the wound 3. ADSCs increased cell proliferation by enhancing the expression of Ki-67 and rPH
STZ-induced rats [108]	Human ADSCs	1. Peri-wound Injection 2. 5 × 10^6^ human ADSCs 3. Immediately after surgery	1. Ulceration contraction rate 2. Histology assessment 3. Vessel density	1. On experimental day 7, the size of foot ulcers in the diabetes transplant group was significantly smaller than that of the non-transplant group; the effect was even more pronounced on day 15 2. Compared with the non-transplant group, the tissue regeneration of the ADSC transplant group was significantly better, and the tissue staining on day 15 showed that the epithelial regeneration of the ADSC transplant group was also better, and the granulation tissue was thicker 3. The vascular density of the ADSC transplant group was also higher than that of the non-transplant group
Zucker diabetic obese rats [109]	Human ADSCs	1. Cell sheet applied to the wound, and artificial skin and adhesive dressings attached to the surface 2. 7 × 10^5^ human ADSCs 3. Immediately after surgery	1. Wound area 2. Histological analysis of wound	1. Compared with the control group, the ADSC transplantation group showed a significant acceleration in wound healing from day 3 2. The mean time for complete wound closure in the ADSC transplantation group was approximately half of that of the control group 3. Histological staining of the wound tissue showed that the dermal thickness in the ADSC transplantation group was significantly higher than that in the control group
STZ-induced rats [110]	Rat ADSCs	1. Cells and pluronic F17 topical gel applied to the wound site 2. 1 × 10^6^ rat ADSCs 3. Immediately after surgery	1. Percentage of wound closure 2. Histology assessment 3. Blood vessel density 4. Cytokine level	1. Starting from day 3, the wound healing speed of the fat-derived stem cell hydrogel group was significantly faster than that of the control group. By day 14, the wound of the fat-derived stem cell hydrogel group was almost completely healed 2. By day 14, the fat-derived stem cell hydrogel group had formed complete granulation tissue, with new hair follicles formed at the wound center. Fibroblast proliferation was observed, collagen deposits were sufficient, and orderly arrangement was found underneath the epidermis 3. By day 14, the blood vessel density of the wound in the fat-derived stem cell hydrogel group was significantly higher than that of the control group 4. The expression of VEGF and TGFβ1 in the fat-derived stem cell hydrogel group was significantly higher than that of the control group
Db/db mice [111]	Ex-4 + Human ADSCs	1. Peri-wound Injection 2. 2.5 × 10^5^ human ADSCs 3. 24 h after surgery	1. Wound healing rate 2. Histology of wound skin 3. Cytokines expression	1. Wound healing in the Ex-4 + ADSC treatment group was significantly faster than the other groups 2. Histological examination of the wound tissue in the Ex-4 + ADSC treatment group showed more prominent endothelial regeneration 4. The expression of VEGF was increased in the Ex-4 + ADSC group compared to the control group, but was lower than when expressed separately
STZ-induced Yorkshire swine [112]	Yorkshire swine ADSCs	1. Peri-wound injection 2. 5 × 10^6^, 10 × 10^6^ Yorkshire swine ADSCs 3. 72 h after surgery, with a 12-h interval	1. Wound closure 2. Histological analysis, 3. mRNA and protein analyses	1. Wound healing in the ADSC treatment group was faster than that in the control group 2. In the ADSC-treated skin samples, intact epidermis with dermal fibrosis and scattered lesions were observed, mainly chronic and occasional foreign body giant cell inflammation, consistent with scar formation 3. RNA analysis showed no significant differences in CD31, nitric oxide synthase, TNF-α and IL-1β, except for a decrease in PDGF in the treatment group; protein analysis showed no significant differences in TGF-β between the treatment and non-treatment groups
STZ-induced Sprague–Dawley rats [113]	Human ADSCs exosomes + EPCs	1. Peri-wound Injection 2. N.M 3. 7 days after surgery	1. Wound area 2. Histology of tissue healing 3. Changes in cytokine factors	1. The EPC + exosome treatment group showed a significant reduction in ulcer area compared to the control group 2. The wound vascular density in the EPC + exosome treatment group was significantly higher than that in the control group 3. Exosomes derived from adipose stem cells reduced glucose-induced EPC aging
STZ-induced Sprague–Dawley rats [114]	Diabetic rat autologous nano-lipid droplets (including ADSCs)	1. Peri-wound injection 2. 2 × 10^5^ ADSCs 3. Immediately after surgery	1. Wound area change 2. Vessel density 3. Angiogenic factor expression	1. Wound healing in the nano-lipid treatment group was significantly faster than that of the control group 2. The endothelial regeneration of the wounds in the nano-lipid treatment group was more complete 3. The capillary density of the granulation tissue in the nano-lipid treatment group was significantly higher than that of the control group 4. The expression of MCP-1 and VEGF was significantly increased in the nano-lipid treatment group
Pressure injury model mice [115]	Human ADSCs	1. PRP + ADSCs injection around the wound 2. 1 × 10^6^ human ADSCs 3. 2 days after surgery	1. Wound healing rate 2. Histology of wounds 3. Immunohistochemical assay	1. Wound healing in the PRP + ADSC treatment group was significantly faster than the other groups 2. The PRP + ADSC treatment group had a lighter wound inflammation reaction compared to the other groups, with a more complete skin structure, and regeneration of appendages was observed 3. More ADSCs gathered in the subcutaneous layer of the edge of the mouse wound
STZ-induced Wistar rats [116]	Rat ADSCs + PBM	1. Peri-wound injection 2. 1 × 10^6^ rat ADSCs 3. Immediately after surgery	1. Wound closure rate 2. Cell amount in the peri-wound area	1. Wound healing in the group treated with PBM and ADSCs was significantly faster than the other groups 2. The number of ulcers on wounds treated with PBM and ADSCs was significantly less than the other groups 3. The quantity of fibroblasts and length of blood vessels in wounds treated with PBM and ADSCs was significantly better than the other groups
STZ-induced mice [117]	Mice ADSCs (normal and diabetic mice)	1. Peri-wound injection 2. 5 × 10^5^ mice ADSCs 3. Immediately after surgery	1. Cell type after injection 2. Cytokine level 3. Wound closure rate	1. Wound healing in the ADSC treatment group was higher than that of the control group, and the healing rate of ADSCs from normal sources was higher than that from diabetic mice 2. ADSCs increased vascular regeneration and collagen expression in wounds of diabetic mice 3. ADSCs reduced the inflammatory response of wounds 4. After injection, ADSCs differentiated into endothelial cells and fibroblasts
Db/db mice [118]	Bcl-2-modified human ADSCs	1. Modified ADSCs and collagen scaffold directly applied to cover the wound 2. N.M 3. 7 days after surgery	1. Wound healing rate 2. Histology assessment 3. Vascularization	1. Wound healing in the ADSC and collagen scaffold treatment group was significantly faster than that of the other control groups 2. The vascular density of granulation tissue in the ADSC and collagen scaffold treatment group was significantly higher than that of the control group 3. Histological analysis performed 1 week after surgery showed that ADSCs maintained high vitality
STZ-induced rats [119]	Rat ADSCs + PBM	1. Peri-wound injection 2. 1 × 10^6^ rat ADSCs 3. N.M	1. Wound healing rate 2. Wound maximum force 3. Mast cell numbers	1. Wound healing of the PBM + ADSC treatment group was significantly faster than that of the other groups 2. The maximum tensile strength of wounds in the PBM + ADSC treatment group was higher than that of the control group 3. The number of mast cells and mast cell granules in the wounds of the PBM + ADSC treatment group decreased significantly
STZ-induced albino rats [120]	Rat ADSCs	1. PRP + ADSCs injection around the wound 2. 2 × 10^6^ rat ADSCs 3. Immediately after surgery	1. Wound area 2. Histological analysis 3. Epidermal thickness 4. Dermal collagen 5. Angiogenesis	1. The combination of ADSCs and PRP improved wound healing through the Notch signaling pathway, with the best wound healing effect observed in this group 2. Histological analysis showed that on the day 7, the wounds in the group treated with ADSCs and PRP had a complete skin structure, abundant collagen fiber status, and thick bundles of collagen in the reticular layer 3. The group treated with ADSCs and PRP showed significantly better epidermal thickness and vascular regeneration in the wound than the other groups
STZ-induced mice [121]	Mice ADSCs	1. Cell sheet cover on the wound 2. 4 × 10^5^ mice ADSCs 3. Immediately after surgery	1. Wound healing rate 2. Histology of wounds	1. Fat stem cell mice accelerated the rate of wound healing in diabetic ulcer mice 2. Adipose stem cells increased the proliferation, migration and regeneration of lymphoendothelial cells through METTL3

*ADSC* adipose-derived stem cells; *EGFP* enhanced green fluorescent protein; *EPC* endothelial progenitor cells; *EX-4* exendin-4; *IL* interleukin; *MCP-1* monocyte chemoattractant protein-1; *N.M.* not mentioned; *PBM* photobiomodulation; *PDGF* platelet-derived growth factor; *PRP* platelet rich plasma; *STZ* streptozotocin; *SVF* stromal vascular fraction; *TGF-β* transforming growth factor-βTNF-α, tumor necrosis factor α; *VEGF* vascular endothelial growth factor; *N.M.* not mentioned

**Table 5:** Clinical trials using ADSCs in diabetic wounds

Patient type	Patient number	Treatment type	Clinical detection	Outcomes	Observed complications/ adverse effects
1. Non-healing ischemic ulcers of the lower extremities ≥ 3cm^2^ 2. Ulcer duration exceeding 3 months 3. High risk of amputation 4. Lower extremity [122]	59	30 × 10^6^Autologous SVF cells peri-wound injection	1. Ulcer size 2. Closure time 3. Blood flow rate 4. Arterial wall elasticity	1. At 6 months, the diabetic foot ulcer closure rate was 100% for 51 subjects, and ≥ 75% for 8 subjects. At 12 months, 100% of the DFUs in 50 subjects had healed, and ≥ 85% had healed in 4 subjects 2. The initial response time to treatment ranged from the 2nd to the 20th week, with an average time of 6.8 weeks. The healing time of ulcer wounds ranged from the 4th to the 32nd week, with an average healing time of 21.6 weeks 3. Among the 33 measured blood vessels, 32 (97%) showed a significant increase in peak systolic velocity, with 11.4% showing changes at the edge of the tibialis anterior muscle, 31.1% showing changes in the more distal dorsalis pedis artery, and 47.8% showing more significant changes in the posterior tibial muscle 4. Among the 33 measured blood vessels, all 33 (100%) showed significant changes in vascular elasticity, with the tibialis anterior artery (92.9%),	The observed adverse reactions are unrelated to the treatment
1. Patients with T1DM and T2DM 2. TcPO2 < 40 mmHg 3. High risk of amputation 4. Lower extremity [123]	9	3.6 ± 0.2 × 10^7^ autologous SVF cells peri-wound injection	1. Changes in TcPO2 values 2. Skin microvascular blood flow levels	1. Before injection of SVF cells, the average value of TcPO2 was 31.3 ± 7.4 mmHg. Twelve weeks after the injection, the TcPO2 value increased to 46.4 ± 8.2 mmHg, and there was a significant improvement compared to the baseline at the 4th and 8th weeks 2. Before injection of SVF cells, the average skin microvascular blood flow level measured was 34.0 ± 21.1 PU. The blood flow level at 12 weeks was 76.1 ± 32.5 PU	There were no adverse events related to SVF cell injection
1. Neural ulcers throughout the layer 2. Disease course > 3 weeks 3. Lower extremity [124]	10	Full-layer dermal cell transplantation based on SVF	1. Rate of change in wound area 2. Thickness and density of dermis and epidermis	1. There was no significant difference between the intervention group and the control group in macroscopic terms, but the intervention group had a better rate of change in wound area than the control group 2. The thickness and density of the subcutaneous vascular layer measured using the skin scanner were significantly higher in the intervention group than in the control group	There were no any side effects from the treatment
1. Patients with T1DM and T2DM 2. Wound area of 1–25cm^2^, and wound depth of Wagner grade I and II 3. Lower extremity [125]	59	Human ADSCs hydrogel	1. Percentage of complete wound healing 2. Average time required for wound healing	1. In week 8, 73% of the treatment group and 47% of the control group achieved complete healing. By week 12, the complete healing rate for the treatment group was 82%, compared to 53% for the control group 2. The median Kaplan–Meier time for complete closure of the wound was 28.5 days for the treatment group and 63.0 days for the control group	There were no serious adverse events related to allogeneic ADSCs treatment
1. Patients with T2DM 2. Wound area of 10–20 cm^2^ 3. Duration of illness > 4 weeks 4. Lesion and wound depth of Wagner grades 1 and 2 5. Lower extremity [126]	20	6 × 10^6^ Human ADSCs injection	1. Wound healing rate 2. Average wound healing time	1. The average healing time for the treatment group was 31.0 ± 10.7 days, while the control group had an average healing time of 54.8 ± 15.0 days 2. Out of 20 lesions, 17 had completely healed wounds, 9 in the treatment group and 8 in the control group	After a mean follow-up of 43.4 ± 8.7 months, there were no adverse events or complications of the ADSCs group
1. patients with diabetic T1DM/TT2DM 2. Longer than 4 weeks for the history of ulcer at screening. 3. Wound size between 1 and 25 cm^2^ 4. Wound depth of Wagner grade I/II 5. Lower extremity [125]	59	1 × 10^6^ allogeneic ADSCs/sheet	1. Wound healing rate 2. Wound healing time	1. 59 patients with diabetic foot ulcers were randomly divided into an ADSCs treatment group and a polyurethane film control group. At week 8, the complete closure rate of wounds in the ADSCs treatment group was 73%, while in the control group was 47%. At week 12, the complete closure rate of wounds in the ADSCs treatment group was 82%, while in the control group was 53%. The median time for wound closure in the ADSCs treatment group and the control group was 28.5 days and 63.0 days, respectively 2. The rate of wound size reduction at week 1 was 49.6 ± 25.7% in the treatment group and 23.0 ± 32.2% in the control group (P = 0.007). The rate of wound size reduction was also statistically significant between the two groups at nine weeks out of 12 study weeks	There were no serious adverse events related to allogeneic ADSCs treatment

*ADSC* adipose-derived stem cells; *N.M.* not mentioned; *SVF* stromal vascular fraction; *T1DM* type 1 diabetes mellitus; *T2DM* type 2 diabetes mellitus; *TcPO2* transcutaneous oxygen pressure

In one study, at 6 months, the diabetic foot ulcer closure rate was 100% for 51 subjects and ≥ 75% for 8 subjects. At 12 months, 100% of the DFUs in 50 subjects had healed, and ≥ 85% had healed in 4 subjects [122]. In another study, 59 patients with diabetic foot ulcers were randomly divided into an ADSCs treatment group and a polyurethane film control group. At week 8, the complete closure rate of wounds in the ADSCs treatment group was 73%, while in the control group was 47%. At week 12, the complete closure rate of wounds in the ADSCs treatment group was 82%, while in the control group was 53%. The median time for wound closure in the ADSCs treatment group and the control group was 28.5 days and 63.0 days, respectively [125]. The healing time of wounds in the group receiving allogeneic ADSC injection was 31 days, which was significantly shorter than that of the control group [126].

According to existing research, there are several possible mechanisms proposed for ADSCs therapy in the treatment of diabetic wounds. ADSCs have paracrine function and can secrete various cytokines, such as VEGF, fibroblast growth factor2, keratinocyte growth factor, TGF-β, platelet-derived growth factor, HGF, and collagen [127]. ADSCs also have the ability to directly differentiate into epithelial components and endothelial cells, playing an important role in dermal remodeling and wound healing [128]. In addition, ADSCs can inhibit the inflammatory response in diabetic wounds through paracrine function. After applying ADSCs, the expression of IL-6, IL-8 [129], and TNF-α [112] in diabetic wounds is significantly downregulated, and inflammatory cell infiltration is reduced [102].

Existing studies have confirmed that ADSCs can promote regulation, neovascularization, and fibrosis, and can be used as a potential therapy for the treatment of diabetic wounds. However, there are still relatively few clinical experiments on the application of ADSCs in the human body [122–126]. Further research is needed to determine more efficient methods of utilizing ADSCs for the treatment of trauma or surgical wounds in diabetic patients, in order to achieve the goal of treating diabetic wounds.

### Diabetic retinopathy

Diabetic retinopathy is a microvascular disease of the retina caused by retinal ischemia [130]. Increasing evidence suggests that diabetes-related neurodegeneration occurs prior to retinal vascular endothelial changes, indicating that diabetic retinopathy should be considered a neurovascular degenerative disease [131, 132].

The self-renewal ability of pericytes and endothelial cells in the eyes of patients with diabetes is impaired, and the repair ability of these two cell types is continuously depleted [133]. Subsequently, the blood flow of capillaries decreases, resulting in hypoxia in adjacent areas of the retina. This hypoxic environment causes upregulation of VEGF, leading to increased vascular permeability [134] and the development of diabetic macular edema, ultimately resulting in loss of visual function [135]. Continuous hyperglycemia leads to abnormal function of ganglion cells, resulting in changes in retinal electrical activity before vascular endothelial changes occur [136]. In addition, hypoxia-inducible factor-1α is induced by hypoxia in the retina, which increases the expression of VEGF regulated by hypoxia, causing intraretinal microvascular abnormalities in the retina [137]. Proliferation and migration of vascular endothelial cells can eventually lead to the formation of neovascularization in the retina, characterized by proliferative diabetic retinopathy.

Currently, the primary treatment for diabetic retinopathy is still aimed at controlling blood sugar to slow down the progression of the disease [138–141]. When the disease progresses to macular edema or proliferative diabetic retinopathy that threatens vision, laser therapy can be used clinically to destroy the surrounding retina and reduce oxygen demand to help alleviate the disease [142]; however, laser therapy may cause many complications, such as decreased visual acuity, thickening of the retina, and loss of visual field [143].

Recently, some studies explored a new method for treating diabetic retinopathy by using ADSCs. This method is based on the ability of ADSCs to differentiate into pericytes, which can prevent neurovascular damage and promote the regeneration of damaged retinas, thereby achieving treatment for diabetic retinopathy [144, 145].

Thomas A. Mendel et al. founded in animal experiments that ADSCs injected into the vitreous body of OIR mice can differentiate into pericytes and integrate into retinal blood vessels, delaying the breakdown of the blood-retinal barrier. After two months of injection, approximately 80% of capillary loss can be prevented. Injection of ADSCs before vascular instability in OIR mice can reduce capillary loss by approximately 50% [146]. In another study, the histopathology of retinal tissue in T1DM nude mice showed a significant reduction in vascular leakage and apoptosis of retinal vascular cells around the eyes that received ADSCs injection compared to those that received saline injection. Additionally, the expression of inflammatory genes related to diabetic retinopathy was downregulated. Furthermore, in vitro experiments confirmed that co-culturing ADSCs with retinal endothelial cells can improve the survival rate of endothelial cells. These findings suggest that ADSCs have a protective effect against retinal damage caused by diabetes [147].

Overall, based on current perspectives, ADSCs can be a potential method for the future treatment of diabetic retinopathy. However, determining the optimal transplantation method and localization of ADSCs remains a challenge. Current research mainly focuses on local or intravenous injection of ADSCs, and further studies are needed to ensure the accurate location of cells in the damaged retinal area after injection into the eye. Additionally, although current research suggests that adipose-derived stem cells can protect retinal blood vessels, the specific therapeutic mechanisms are still unclear and require further investigation. Furthermore, the current research on the use of ADSCs for the treatment of diabetic retinopathy is limited to animal experiments, and there is still a long way to go before ADSCs can be used as an actual treatment method.

### Diabetic nephropathy

Diabetic nephropathy (DN) is the leading cause of end-stage renal disease and the main cause of death for patients with T1DM and T2DM [148]. The main feature of DN is the abnormality of kidney function and morphology. Abnormalities in the morphology of glomeruli include increased glomerular size, podocyte injury, gradual accumulation of ECM, mesangial matrix expansion, thickening of the glomerular basement membrane, and the appearance of glomerulosclerosis and interstitial fibrosis. Functional abnormalities include proteinuria, decreased glomerular filtration rate, and increased glomerular perfusion and filtration [149]. Long-term high blood sugar, high blood pressure, and local inflammation can lead to progressive and irreversible damage to the glomeruli and renal tubulointerstitium, ultimately resulting in renal dysfunction and eventually progressing to renal failure [148].

Under special conditions, podocytes and mesangial cells release various mediators to promote functional and morphological changes in glomeruli [150]. Mediators such as VEGFA, TGF-β1, angiotensin II, angiotensin-converting enzyme, inflammatory cytokines, and glomerular capillary remodeling cytokines can induce pathological changes in the kidneys. They activate cell remodeling signaling pathways, increase ECM synthesis, or activate NADPH oxidase, leading to increased oxidative stress levels. These changes can alter cell morphology and contribute to the development of kidney disease [151]. Furthermore, persistent hyperglycemia can generate advanced glycation end products (AGEs) in plasma and tissues, which can exacerbate DN via two mechanisms. AGEs can bind to matrix proteins like laminin and type IV collagen, inhibiting their breakdown by matrix metalloproteinases. This leads to an accumulation of excessive ECM proteins and fibrosis [152]. AGEs can bind to receptors on podocytes and mesangial cells, causing the secretion of fibrosis-promoting factors like VEGF, connective tissue growth factor, and TGF-β1, and an increase in NADPH oxidase expression. These factors promote the proliferation, expansion, and hypertrophy of glomerular cells [153]. Inflammation plays a significant role in the development of DN. As glomerular function deteriorates, inflammatory cells infiltrate the renal interstitium and release factors that worsen the progression of DN, including TNF-α, IFN-γ, IL-1, IL-6, and MCP-1. Inflammatory cells can also activate NADPH oxidase, leading to local oxidative stress responses [154].

Increasing evidence suggests that exosomes derived from stem cells are relatively safe and effective in treating kidney diseases in rat or mouse models [155]. The exosomes secreted by MSCs play a significant protective role in acute kidney injury and chronic kidney disease [156]. Exosomes are nanoscale membrane vesicles released by various types of cells, including mesenchymal stem cells [157]. The microRNAs can be enclosed in exosomes and serve as potential paracrine regulatory factors involved in the regulation of many diseases, such as ischemic diseases and degenerative eye diseases [158, 159]. The microRNAs produced by MSCs, such as miR-150 and miR-134, play a crucial role in the treatment of DN [160]. Exosomes secreted by human urine-derived stem cells alleviate DN and high glucose-induced podocyte injury through the transfer of miR16-5p [161]. Therefore, some researchers have attempted to use ADSCs-Exos to achieve the goal of treating DN, and have successfully improved the functional impairments of foot cells and symptoms of DN to varying degrees.

In vivo studies have also shown that ADSC-Exo can inhibit high glucose-induced podocyte apoptosis in mice [162]. According to the research of Duan Y et al., exosomes produced by ADSCs contain miR-26a-5p, which can be transferred to glomerular podocytes and improve DN in diabetic mice. In vitro studies have shown that ADSCs-Exo-miR-26a-5p can prevent podocyte apoptosis caused by high glucose by targeting TLR4, reducing the expression of VEGFA, inhibiting the pathway of NF-κB, and suppressing oxidative stress reactions [163]. In the experiment conducted by Jin et al. ADSC-Exo-miR-486 can inhibit high glucose-induced podocyte apoptosis by targeting Smad1, downregulating its expression, and suppressing the mTOR pathway which promotes autophagy flux and reduces podocyte apoptosis [162]. Besides they also founded that ADSCs-Exo-miR-215-5P can inhibit the expression of zinc finger E-box-binding homeobox 2, alleviate the progression of epithelial-mesenchymal transition, and foot cell migration [164].

In summary, according to the current results, ADSCs-Exo have potential therapeutic effects in the treatment of diabetic nephropathy, and in the future, they may be a relatively good choice for the treatment of DN. However, there are still some issues that need to be addressed, such as optimizing the preparation methods of ADSC extracellular vesicles, determining the active molecules in the extracellular vesicles, and exploring methods to accurately deliver the extracellular vesicles to the kidneys. These will contribute to the translation of adipose-derived stem cells into clinical applications.

Recent studies have shown that liver changes is another complication of DM [165]. Hyperglycemia caused by DM increases the risk of liver damage and liver fibrosis [166], severely affecting the health and quality of life of patients. DM is closely associated with liver diseases [165, 167], but the pathological and physiological basis and progression of liver changes in DM are not yet fully understood, and effective early intervention is lacking. Some studies have attempted to transplant ADSCs into animals to alleviate diabetes-induced liver damage and fibrosis, and have achieved certain positive results [168, 169]. This provides an important theoretical and experimental basis for further research and development of ADSCs for the treatment of DM-related liver diseases. However, clinical trials have not yet been conducted, and the explanation of the therapeutic mechanisms and pathways of ADSCs is not sufficiently detailed, requiring further research for clarification.

**Fig. 2:**
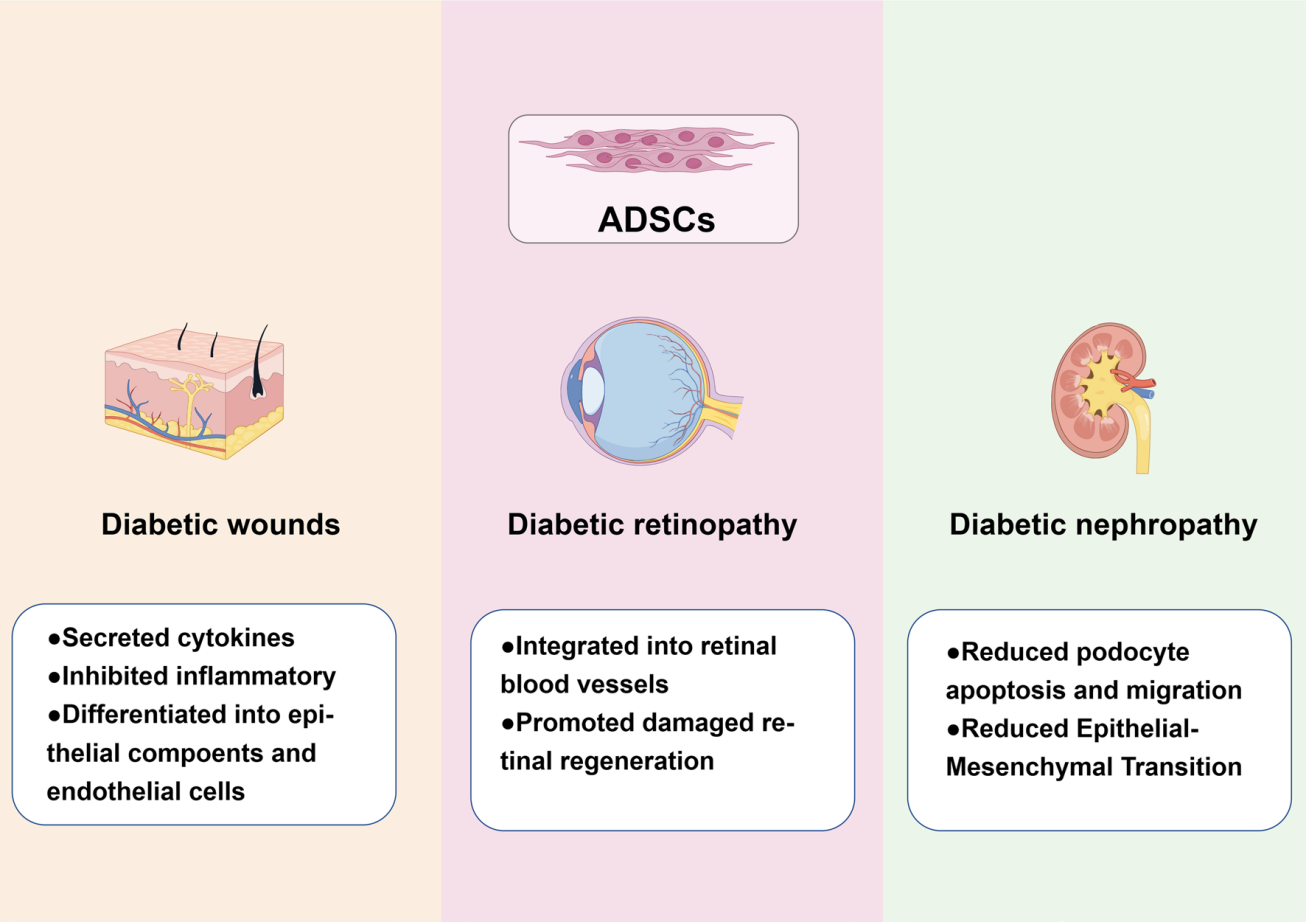
The application and mechanism of ADSCs in the complications of Diabetes Mellitus. *ADSCs* adipose-derived stem cells. All of the elements in the diagram were provided by Figdraw (http://www.fgdraw.com)

The potential mechanisms of ADSCs application in complication of DM are summarized in Fig. 2.

## Current challenges

Although ADSCs have broad prospects for application in disease treatment and tissue engineering, their application still faces some challenges. The following are some possible issues:

*Complications issues* There are certain difficulties and risks in obtaining and processing adipose tissue, such as the risk of wound infection due to improper handling and the possibility of blood clots from excessive intravenous infusion of ADSCs. Further research and clinical observation are needed to ensure the long-term effectiveness and safety of ADSCs therapy.

*Standardization issues* ADSCs are derived from various tissues, such as subcutaneous adipose tissue, breast tissue, and bone marrow. The differences in preparation and culture conditions of ADSCs mean that it is not guaranteed to obtain the same cell population in different laboratories. ADSCs from different sources have differences in biological characteristics, differentiation ability, and immunogenicity, which poses a challenge to the stability of ADSCs application. ADSCs have different abilities and functions, and standardized methods have not been established. More researches are needed to develop good quality control standards to ensure the consistency and stability of cells and achieve the desired therapeutic effects. In addition, it is also necessary to determine the number of cells needed for transplantation to cure diabetes mellitus and its complications in order to reduce the number of transplantations and patient suffering.

*Transplantation efficiency issues* During the in vitro culture process, some ADSCs may be lost, and similarly, some ADSCs may be lost during the transplantation process, which may affect the transplantation effect. The survival rate of adipose-derived stem cells after transplantation is an important issue, and optimization of long-term preservation and storage conditions of ADSCs needs to be addressed. More effective methods need to be explored to ensure the purity and stability of cells during the cell culture and expansion process.

*Plasticity issues* ADSCs need to undergo differentiation to generate insulin-secreting cells. ADSCs may be unstable during the differentiation process, leading to inconsistent results in differentiation products. The efficiency and stability of the differentiation process are key issues, therefore, further research and exploration are needed to optimize the stability of ADSCs differentiation.

It should be noted that the above issues are just some potential challenges mentioned in this article, and there may be other issues in actual applications.

## Conclusions

In summary, an increasing amount of research suggests that ADSCs may serve as a new therapeutic approach for DM. Treatment with ADSCs has the potential to improve high blood glucose levels and alleviate symptoms of related complications in both animals and humans. However, there is still much work to be done in order to translate ADSCs into practical clinical applications. Further research and clinical observation are needed to assess the long-term effects of ADSCs treatment and minimize potential risks associated with their usage, in order to achieve more reliable and effective benefits in future clinical applications.

## Data Availability

Data sharing is not applicable to this article, as no datasets were generated or analyzed during the current study.

## Abbreviations

ADSCs: Adipose-derived stem cells
AGE: Advanced glycation end products
AMPK: AMP-activated protein kinase
BM-MSC: Bone marrow-derived MSCs
DFU: Diabetic foot ulcer
DM: Diabetes mellitus
DN: Diabetic nephropathy
ECM: Extracellular matrix
GLP-1: Glucagon-like peptide-1
HGF: Hepatocyte growth factor
HLA: Human leukocyte antigen
IFN-γ: Interferon-gamma
IL: Interleukin
INSR: Insulin receptors
IPC: Insulin-producing cells
MSC: Mesenchymal stem cell
PDX1: Pancreatic and duodenal homeobox 1
PI3K: Phosphatidylinositol 3-kinase
STZ: Streptozotocin
T1DM: Type 1 diabetes mellitus
T2DM: Type 2 diabetes mellitus
TGF-β: Transforming growth factor-β
Th1: T helper 1
TIMP-1: Tissue inhibitor of metalloproteinase 1
TNF-α: Tumor necrosis factor-alpha
vWF: Von Willebrand factor
